# Editorial: 150 years of laryngectomy: reviews on the state of the art and future perspectives

**DOI:** 10.3389/fonc.2025.1664111

**Published:** 2025-08-08

**Authors:** Dietmar Thurnher, Andreas Dietz, Cesare Piazza

**Affiliations:** ^1^ Department of Otorhinolaryngology, Medical University of Graz, Graz, Austria; ^2^ Department of Otorhinolaryngology, Medical University of Leipzig, Leipzig, Germany; ^3^ Department of Otorhinolaryngology, Universita degli studi di Brescia, Brescia, Italy

**Keywords:** total larygogectomy, partial laryngectomy, head and neck cancer, transoral laser and robotics surgery, medical history

In 1873, Theodor Billroth performed the world’s first total laryngectomy in a human, a radical procedure that would forever change the landscape of head and neck oncology. At a time when surgical interventions for cancer were limited and often fatal, Billroth’s daring operation—removing the entire larynx—offered a new possibility for patients with advanced laryngeal cancer. Over the past 150 years, this procedure has not only endured but evolved, reflecting both the progress and the persistent challenges in the field of surgical and nonsurgical oncology, which is described in detail in this Research Topic.

In their paper *Thurnher* et al. provide an historical overview of the surgical developments of total laryngectomy of the last 150 years from a desperate measure for patients to a cornerstone of treatment for advanced laryngeal malignancies. Despite the rise of organ-preserving treatments like radiochemotherapy, total laryngectomy remains crucial for advanced cases or as salvage surgery when other therapies fail. The review not only discusses the evolution and current role of total laryngectomy but also offers a comparative analysis with other surgical approaches, including open partial laryngectomies and minimally invasive techniques like transoral laser or robotic resections. Additionally, it contrasts these surgical methods with non-surgical alternatives, such as radiation therapy and combined radiochemotherapy, highlighting the nuanced decision-making required in the treatment of laryngeal cancer.

Despite remarkable advances in surgical technique and perioperative care, total laryngectomy continues to be associated with significant complications like pharyngocutaneous fistulas (PCF), which is the topic of the review by (Piazza et al.). PCF remains one of the most frequent and serious complications following the procedure, occurring in approximately 10% of patients despite technical improvements. The strategies for preventing and managing PCF is detailed in the review and include various reconstructive techniques using both pedicled and free flaps and additional preventive measures like the simultaneous use of salivary bypass tubes, optimization of perioperative nutrition, control of comorbidities, rational antibiotic use, and careful selection of closure techniques.

Dietz et al. describe in their review the emergence of “larynx organ preservation” (LOP) strategies which have altered the treatment landscape for laryngeal cancer. These non-surgical approaches, primarily consisting of simultaneous chemoradiation or neoadjuvant chemotherapy followed by radiotherapy, offer the compelling possibility of tumor eradication while preserving the patient’s natural voice and swallowing function.

The distinction between good and bad candidates for LOP remains unclear, leading to treatment decisions that often depend more on patient preferences, surgeon experience, and institutional philosophy than on clear clinical guidelines. Furthermore, a significant proportion of patients ultimately require salvage surgery due to tumor persistence after treatment. However, the introduction of immune check point inhibitors in a neoadjuvant setting has shown promising results in current trials which has the potential of a paradigm shift in the treatment of head and neck cancer.

Despite advances in multimodal treatment approaches, patient outcome with laryngeal cancer remains disappointing, particularly for those with high-risk locally advanced disease and recurrent or metastatic cases. Fuereder et al. discuss in their review current efforts to improve outcome and quality of life of this patient population. Traditional treatment intensification through induction chemotherapy has failed to deliver meaningful improvements in overall survival, though it may offer some benefit for larynx preservation in select patients. Recent research efforts have focused on integrating innovative immunotherapies into established treatment protocols. Perioperative immunotherapy regimens show promise for locally advanced cases, offering the dual advantage of preserving laryngeal function while optimizing both event-free survival and overall survival outcomes.

Billroth’s legacy

Today, as we mark more than 150 years since Billroth’s pioneering operation, total laryngectomy continues to serve as both a primary treatment for locoregionally advanced disease and a salvage procedure for recurrent cancer. While organ-preserving therapies have revolutionized the management of laryngeal cancer, the fundamental principle established by Billroth—that aggressive surgical intervention can be life-saving—remains as relevant today as it was in 1873.

